# Effects of a low-carbohydrate diet on glycemic control in outpatients with severe type 2 diabetes

**DOI:** 10.1186/1743-7075-6-21

**Published:** 2009-05-06

**Authors:** Hajime Haimoto, Tae Sasakabe, Kenji Wakai, Hiroyuki Umegaki

**Affiliations:** 1 Department of Internal Medicine, Haimoto Clinic, 1-80 Yayoi, Kasugai, Aichi 486-0838, Japan; 2 Department of Clinical Nutrition, Haimoto Clinic, 1-80 Yayoi, Kasugai, Aichi 486-0838, Japan; 3 Department of Preventive Medicine/Biostatistics and Medical Decision Making, 65 Tsuruma, Showa, Nagoya, Aichi 466-8550, Japan; 4 Department of Geriatrics, Nagoya University Graduate School of Medicine, 65 Tsuruma, Showa, Nagoya, Aichi 466-8550, Japan

## Abstract

We previously demonstrated that a loosely restricted 45%-carbohydrate diet led to greater reduction in hemoglobin A1c (HbA1c) compared to high-carbohydrate diets in outpatients with mild type 2 diabetes (mean HbA1c level: 7.4%) over 2 years. To determine whether good glycemic control can be achieved with a 30%-carbohydrate diet in severe type 2 diabetes, 33 outpatients (15 males, 18 females, mean age: 59 yrs) with HbA1c levels of 9.0% or above were instructed to follow a low-carbohydrate diet (1852 kcal; %CHO:fat:protein = 30:44:20) for 6 months in an outpatient clinic and were followed to assess their HbA1c levels, body mass index and doses of antidiabetic drugs. HbA1c levels decreased sharply from a baseline of 10.9 ± 1.6% to 7.8 ± 1.5% at 3 months and to 7.4 ± 1.4% at 6 months. Body mass index decreased slightly from baseline (23.8 ± 3.3) to 6 months (23.5 ± 3.4). Only two patients dropped out. No adverse effects were observed except for mild constipation. The number of patients on sulfonylureas decreased from 7 at baseline to 2 at 6 months. No patient required inpatient care or insulin therapy. In summary, the 30%-carbohydrate diet over 6 months led to a remarkable reduction in HbA1c levels, even among outpatients with severe type 2 diabetes, without any insulin therapy, hospital care or increase in sulfonylureas. The effectiveness of the diet may be comparable to that of insulin therapy.

## Background

Carbohydrate-restricted diets (CRDs) have been reported to be effective for glycemic control [1-7] in type 2 diabetes (T2DM). We recently demonstrated that a loosely restricted 45%-carbohydrate diet (carbohydrate-reduced diet: CRD) led to a significant reduction in hemoglobin A1c (HbA1c) levels with a tapering off of sulfonylureas compared to a 60%-carbohydrate diet (high-carbohydrate diet: HCD) over 2 years among outpatients with mild T2DM (mean HbA1c = 7.4%) [8].

Little is known about the long-term effects of CRDs on patients with severe T2DM. We therefore tried to determine whether good glycemic control can be achieved with a stricter CRD (30%-carbohydrate), even in outpatients with severe T2DM in an outpatient clinic.

## Patients and methods

We recruited outpatients with T2DM having HbA1c levels of 9.0% or above between September 2005 and September 2007 in Haimoto Clinic, and followed their HbA1c levels, body mass index (BMI) and doses of antidiabetic drugs monthly for 6 months. We also followed their serum lipid profiles, serum creatinine and blood pressure. Patients with serum creatinine levels > 1.5 mg/dl, severe diabetes complications (proliferative retinopathy, symptomatic neuropathy and diabetic foot), ketoacidosis, soft drink ketosis [9] and malignant tumor were excluded. Five patients who developed ketosis received fluid therapy for a few days, and did not require any inpatient care or insulin therapy. We intended to taper the dose of sulfonylureas as soon as the patients' HbA1c levels were controlled, and to prescribe metformin, acarbose and pioglitazone. The patients were instructed to maintain their usual level of physical activity throughout the study. Changes in activity levels were investigated by questionnaire. The study protocol was identical to that of the previous study [8] and was approved by the Ethical Committee of the Nagoya Tokusyukai General Hospital. All patients provided written informed consent.

The main principle of the CRD was to eliminate carbohydrate-rich food twice a day at breakfast and dinner, or eliminate it three times a day at breakfast, lunch and dinner. Table 1 shows the list of foods that the subjects were instructed to avoid. There were no other restrictions. Patients on the CRD were permitted to eat as much protein and fat as they wanted, including saturated fat. Their details were described previously [8]. At the end of the study, dietary intake was assessed based on 3-day food records. Changes in HbA1c and BMI were assessed by the Friedman test, and changes in serum LDL-cholesterol, HDL-cholesterol, triglyceride, creatinine and blood pressure were assessed by the paired *t*-test.

**Table 1:** Carbohydrate-rich foods instructed to remove in the carbohydrate-reduced diet

Staple foods	rice, bread, corn, spaghetti, noodle made of wheat or buckwheat, potato, sweet potato, taro and yam
Fruits	pear, apple, persimmon, mikan, orange, grapefruit, peach, grape, melon, water melon, banana, pine apple and Japanese chestnut, etc.
Vegetables	carrot, Indian lotus, pumpkin and autumn squash
Confectioneries	
Drink	beverages containing sugar, glucose and fructose, and milk
Alcohol	brew: sake, beer and wine (Distilled liquor was not restricted.)

## Results

Thirty-three patients participated in this study. Background characteristics of the patients are shown in Table 2. Two patients (6%) on the CRD dropped out after 4 months. Of the remaining 31 patients, the total energy intake (mean ± SD) was 1852 ± 549 kcal/day (Table 3). The daily average intakes of carbohydrate, fat and protein were 137 ± 41 g (30 ± 10% of total energy), 91 ± 34 g (44 ± 10%) and 91 ± 30 g (20 ± 4%), respectively, with the mean fiber intake being 14 ± 6 g. The carbohydrates were mainly derived from rice and noodles made from wheat or buckwheat, and also from potatoes, fruits, bread and confectioneries.

**Table 2:** Background characteristics in all patients (n = 33)

Age (years)	59 ± 9
Male/Female (n)	15/18
Duration of diabetes (months)	59 ± 9
Body weight (kg)	60.9 ± 11.0
Body mass index	23.8 ± 3.3
Hemoglobin A1c (%)	10.9 ± 1.6
Serum LDL-cholesterol (mg/dl)	147 ± 50
Serum HDL-cholesterol (mg/dl)	51 ± 14
Serum triglyceride (mg/dl)	187 ± 177
Serum creatinine (mg/dl)	0.68 ± 0.20
Systolic blood pressure (mmHg)	138 ± 17
Diastolic blood pressure (mmHg)	81 ± 11
Patients with medications (n)	
Antidiabetic drugs	10
Antihypertensive drugs	11
Lipid-lowering drugs	4

Plus-minus values indicate means ± SD

**Table 3:** Daily dietary intake at 6 months from baseline (n = 31)

Total energy intake (kcal)	1852 ± 549
Carbohydrate (g)	137 ± 41
Fiber (g)	14 ± 6
Carbohydrate (% energy)	30 ± 10
Fat (g)	91 ± 34
Saturated fat (g)	26 ± 13
Monounsaturated fat (g)	35 ± 16
Polyunsaturated fat (g)	17 ± 7
Fat (% energy)	44 ± 10
Protein (g)	91 ± 30
Protein (% energy)	20 ± 4
Foods rich in carbohydrate (g)	
Rice	132 ± 86
Noodles	25 ± 43
Bread	15 ± 29
Potatoes	11 ± 18
Fruit	39 ± 54
Confectioneries	12 ± 20
Sugar	7 ± 6

Plus-minus values indicate means ± SD.

Two drop-out patients are excluded.

**Figure 1:**
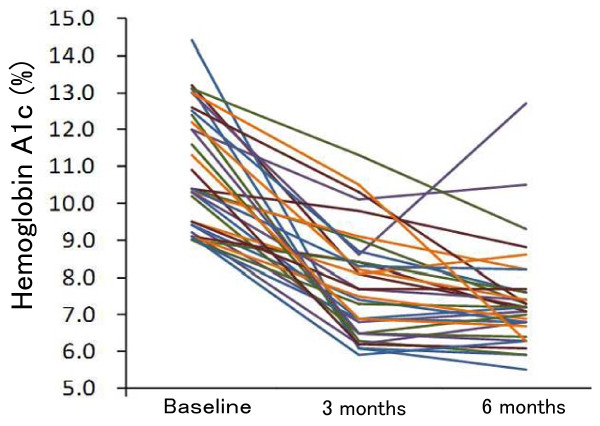
**Individual changes in hemoglobin A1c over 6 months (n = 33)**. Two drop-out patients were included.

**Figure 2:**
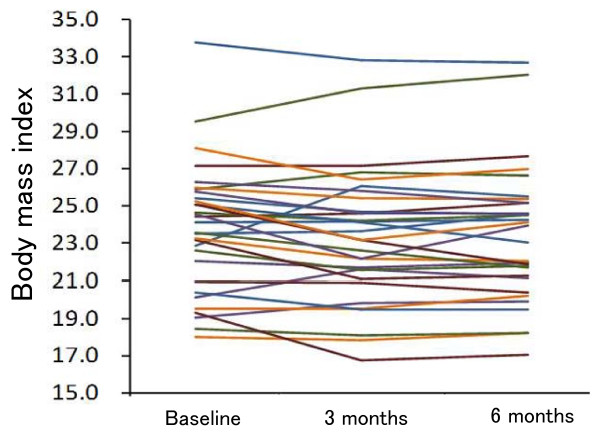
**Individual changes in body mass index over 6 months (n = 33)**. Two drop-out patients were included.

The mean HbA1c level decreased sharply from baseline (10.9 ± 1.6%) to 7.8 ± 1.5% at 3 months, and then more gradually to 7.4 ± 1.4% at 6 months (*P *< 0.001) (Table 4 and Figure 1). BMI slightly decreased over 6 months, but the decrease did not reach statistical significance (*P *= 0.057) (Table 4 and Figure 2). HbA1c levels of the two drop-out patients were 13.0% and 9.5% at baseline, which decreased to 8.6% and 8.1% after 3 months but returned to 12.6% and 8.6% after 6 months, respectively. When the two patients were excluded, the mean HbA1c level after 6 months was 7.2 ± 1.0%. No adverse effect was observed except for mild constipation. One female patient had an increased physical activity level during the study period in spite of our instructions. However, her increase in physical activity was no more than one hour of walking per day, four days a week. She had implemented an 11%-carbohydrate diet without any antidiabetic drug, and her HbA1c level decreased from 14.4% at baseline to 6.1% after 3 months and had been maintained at 5.5% after 6 months.

**Table 4:** Changes in HbA1c, BMI, serum lipids and creatinine, blood pressure and dose of antidiabetic drugs (n = 33)

	Baseline	After 3 months	After 6 months	*P*
HbA1c (%)	10.9 ± 1.6 (9.0 – 14.4)	7.8 ± 1.5 (5.9 – 11.3)	7.4 ± 1.4 (5.5 – 12.7)	< 0.001
BMI	23.8 ± 3.3 (18.0 – 33.8)	23.5 ± 3.4 (16.8 – 32.8)	23.5 ± 3.4 (17.1 – 32.7)	0.057
Serum LDL-cholesterol (mg/dl)*	142 ± 50 (51 – 260)		128 ± 34 (61 – 213)	0.036
Serum HDL-cholesterol (mg/dl)*	52 ± 14 (30 – 77)		59 ± 16 (34 – 94)	0.008
Serum triglyceride (mg/dl)*	182 ± 189 (50 – 823)		157 ± 178 (34 – 721)	0.39
Serum creatinine (mg/dl)	0.68 ± 0.20 (0.38 – 1.20)		0.71 ± 0.21 (0.44 – 1.25)	0.21
Systolic BP (mmHg)	138 ± 17 (104 – 175)		135 ± 14 (97 – 157)	0.24
Diastolic BP (mmHg)	81 ± 11 (59 – 102)		81 ± 11 (48 – 113)	0.64
Patients with antidiabetic drugs (n)				
Glibenclamide	n = 6 (4.0 mg)	n = 3 (1.7 mg)	n = 1 (2.5 mg)	
Glimepiride	n = 1 (3.0 mg)	n = 2 (0.75 mg)	n = 0 (0 mg)	
Tolbutamide	n = 0 (0 mg)	n = 0 (0 mg)	n = 1 (500 mg)	
Metformin	n = 0 (0 mg)	n = 2 (500 mg)	n = 5 (600 mg)	
Nateglinide	n = 2 (135 mg)	n = 2 (180 mg)	n = 0 (0 mg)	
Pioglitazone	n = 0 (0 mg)	n = 0 (0 mg)	n = 2 (30 mg)	
Miglitol	n = 1 (150 mg)	n = 3 (100 mg)	n = 7 (125 mg)	

HbA1c: hemoglobin A1c; BMI: body mass index; BP: blood pressure. Values are means ± SD. The numbers in parenthesis in HbA1c, BMI, serum lipids, serum creatinine and blood pressure indicate ranges, and those in antidiabetic drugs show a mean daily dose. Two drop-out patients are included in the analysis.

*: Four patients are excluded from serum lipids analysis because they received lipid-lowering drugs.

Ten patients had already been prescribed antidiabetic drugs by other physicians at baseline. The number of patients who were on sulfonylureas decreased over 6 months (glibenclamide: from 6 to 1, glimepiride: from 1 to 0, tolbutamide: from 0 to 1) (Table 4). Of the 31 patients, 12 received other antidiabetic drugs at the end of the study; a relatively low dose of metoformin or miglitol was mainly prescribed. No patient required inpatient care or insulin therapy.

**Figure 3:**
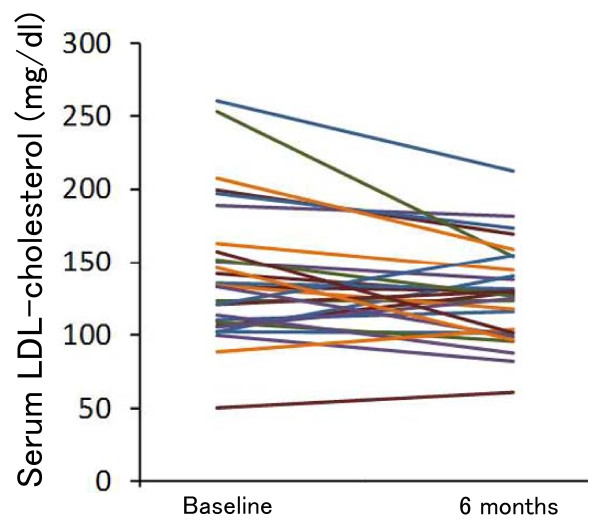
**Individual changes in serum LDL-cholesterol concentrations over 6 months (n = 29)**. Four patients were excluded because they received lipid-lowering drugs.

**Figure 4:**
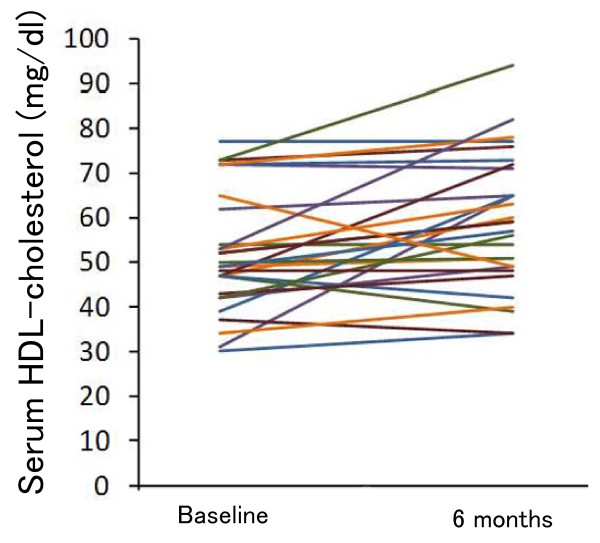
**Individual changes in serum HDL-cholesterol concentrations over 6 months (n = 29)**. Four patients were excluded because they received lipid-lowering drugs.

Excluding 4 patients who were prescribed the lipid-lowering drugs during the study period, the mean serum LDL-cholesterol levels of the subjects decreased (*P *= 0.036) (Table 4 and Figure 3), while their mean HDL-cholesterol levels increased (*P *= 0.008) over 6 months (Table 4 and Figure 4). The mean serum triglyceride concentrations decreased over 6 months, but the change did not reach statistical significance (*P *= 0.39) (Table 4). We found no significant change in serum creatinine. Eleven patients were prescribed antihypertensive drugs during the study period. No significant change was detected in systolic and diastolic blood pressure.

## Discussion

This study demonstrated that good glycemic control with a tapering off of sulfonylureas and improvement of the serum cholesterol profile can be achieved with a 30%-carbohydrate diet among outpatients with severe T2DM at an outpatient clinic. The mean HbA1c level decreased by 1.0% per month during the first 3 months. Our results must be cautiously interpreted due to the lack of a control group. However, it can be difficult to decrease HbA1c by 3.5% by means of HCDs over 6 months without any inpatient care, insulin therapy or reinforcement of sulfonylureas. When HCDs were incorporated into the dietary therapy for severe T2DM, the expected decreases in HbA1c levels for the insulin therapy ranged from 1.5% to 2.5%, while that for oral sulfonylureas was about 1.5% in T2DM [10]. Moreover, practitioners in Japan have achieved HbA1c levels ranging from 7.6 to 7.7% by means of insulin treatment or insulin plus oral antidiabetic drugs among patients with severe T2DM [11]. Thus, the effectiveness of the 30%-carbohydrate diet could be compared to that of the insulin therapy with HCDs, so that CRD can be considered an alternative to conventional HCDs in the dietary management of T2DM.

When total energy intake is strictly restricted, there may be little difference in glycemic control between CRDs and HCDs [12]. In contrast, CRD has the advantage that is does not restrict fat and protein and does not require explicit attention to total energy intake [8], giving rise to a low attrition rate of 6% over 6 months. Beyond calorie restriction, CRD has an advantage in comparison to HCDs, in that dietary fat and protein have little effect on postprandial blood glucose levels, while dietary carbohydrate is a major stimulus [3,4].

The attrition rate for CRD was lower than that in other studies on CRDs (22% for 3 months, and 45%, 33% and 27% for 6 months) [7,12-14]. It should also be noted that the attrition rate in the previous study on CRD was lower in the first year (8%) but higher in the second year (33%) [8]. This is probably due to reduced motivation because of the improvement of glycemic control during the first year.

In conclusion, the 30%-carbohydrate diet led to a remarkable reduction in HbA1c levels from baseline to 6 months, together with improvement of serum cholesterol levels without any insulin therapy, hospital care or reinforcement of sulfonylureas, even among outpatients with severe T2DM. The effectiveness of the diet may be comparable to that of insulin therapy. CRD could be implemented with good compliance among outpatients; therefore, it can be an alternative to HCDs in the dietary management of T2DM.

## Abbreviations

BMI: Body mass index; CRDs: Carbohydrate-restricted diets; CRD: Carbohydrate-reduced diet; HbA1c: Hemoglobin A1c; HCD: High-carbohydrate diet; T2DM: Type 2 diabetes.

## Competing interests

The authors declare that they have no competing interests.

## Authors' contributions

HH and TS designed the study and participated in data collection. HH, KW and HU performed statistical analysis and interpretation. HH wrote the manuscript.
